# Glucose levels as a prognostic marker in patients with ST-segment elevation myocardial infarction: a case–control study

**DOI:** 10.1186/s12902-016-0108-8

**Published:** 2016-06-01

**Authors:** Victoria Karetnikova, Olga Gruzdeva, Evgenya Uchasova, Anastasia Osokina, Olga Barbarash

**Affiliations:** Federal State Budgetary Institution “Research Institute for Complex Issues of Cardiovascular Diseases”, Kemerovo, the Russian Federation; Federal State Budget Educational Institution of Higher Professional Education “Kemerovo State Medical Academy” the Ministry of Health of the Russian Federation, Kemerovo, the Russian Federation

**Keywords:** Myocardial infarction, Diabetes mellitus, Blood glucose, Prognosis

## Abstract

**Background:**

Patients with myocardial infarction (MI) have a high mortality. Therefore, new risk markers and predictors of an adverse outcome for MI are required. The role of hyperglycemia in the development of cardiovascular complications in MI patients is still unclear.

**Methods:**

A total of 529 consecutive patients with the diagnosis of ST-segment elevation acute coronary syndrome within 24 h of the onset of symptoms were included in the study. All of the patients underwent blood glucose measurement at admission to hospital. The glycemic profile, including measurement of blood glucose levels early in the night and in the morning (3 a.m. and 5 a.m.), was assessed in 77 patients with diabetes on days 6–10 of the course of MI to monitor the efficiency of blood glucose-lowering therapy and to detect hypoglycemic episodes.

**Results:**

In-hospital mortality showed relationship between the level of blood glucose on admission and in-hospital mortality in patients with MI with ST-segment elevation in combination with diabetes mellitus. There was a direct linear relationship between blood glucose levels and in-hospital mortality in patients without diabetes.

**Conclusion:**

Episodes of hypoglycemia recorded in MI patients with diabetes in the hospital stage of treatment do not determine the prognosis, but enable identification of patients with an unfavorable course in the postinfarction period.

## Background

The role of hyperglycemia in the development of cardiovascular complications in myocardial infarction (MI) patients is still a matter of concern. There are two major perspectives on the role of hyperglycemia in the medical literature. One hypothesis is that hyperglycemia in patients with acute coronary syndrome (ACS) is induced by activation of adrenergic receptors. Another hypothesis is that hyperglycemia in patients with MI is a marker of pre-existing, but as yet undetected, carbohydrate metabolism disorders [1]. Recent studies have shown that hyperglycemia is often overlooked in 66 % of patients admitted with acute MI, even though it meets the criteria for diabetes mellitus (DM) [2]. An association between high glucose levels in ACS patients and increased overall mortality has been shown [3]. Elevated blood glucose levels in ACS patients without DM are considered to be associated with a less favorable prognosis than in diabetic patients [4].

Hypoglycemia induced by adrenergic stimulation causes recurrent myocardial ischemia and triggers ventricular arrhythmias. Moreover, hypoglycemia inhibits metabolic processes in the myocardium and induces apoptosis in cardiomyocytes. The increased release of anti-insulin hormones results in the development of hyperglycemia, especially in patients suffering from short-course DM [5]. Therefore, alterations in blood glucose levels in patients with MI, regardless of diabetic status in the in-hospital period, may be regarded as a metabolic marker or a predictor of adverse outcomes.

The present study aimed to assess the role of hyper- and hypoglycemia in in-hospital and 1-year prognoses in patients with ST-segment elevation MI.

## Methods

A total of 529 consecutive patients who were admitted to the Kemerovo Cardiology Dispensary in 2009 with a diagnosis of ST-segment elevation MI within 24 h of the onset of symptoms were included in the study. The exclusion criteria were as follows: MI complicating percutaneous coronary intervention or coronary artery bypass grafting surgery, end-stage renal disease (glomerular filtration rate <30 mL/min), a positive history of diabetic coma, cancer pathology, and other diseases that greatly reduce life expectancy. 41 (7.9 %) patients revealed heart failure before, 124 (23.9 %) had previous MI, 213 (41.0 %) smoked.

The study protocol was approved by the local ethics committee of the Federal State Budgetary Institution “Research Institute for Complex Issues of Cardiovascular Disease”, developed in accordance with the WMA Declaration of Helsinki “Ethical principles for medical research involving human subjects” (amended in 2000) and “Rules for clinical practice in the Russian Federation”, approved by the Ministry of Health of the Russian Federation on 19 June 2003. The study protocol was approved by the Local Ethics Committee of the Kemerovo Cardiology Dispensary. All patients provided written informed consent prior to their participation in the study.

ST-segment elevation MI was diagnosed according to the Russian Society of Cardiology (2007) as follows: chest pain lasting more than 20 min; ST-segment elevation on electrocardiogram in two or more contiguous leads with the cut-off points of ≥0.1 mV or complete left bundle branch block; elevated troponin T levels ≥0.1 ng/mL and/or creatinine kinase-MB levels >25 IU/L. The presence of at least two criteria, including elevated biochemical markers of myocardial necrosis, was considered sufficient for diagnosis of ST-segment elevation MI.

Repeated measurement of blood glucose levels and an oral glucose tolerance test (OGTT), performed on days 10–14 in the in-hospital period, were used to diagnose type 2 DM in patients who had unrecognized diabetes. A positive history of DM was confirmed by examination of medical records.

Diabetes was diagnosed when two or more independent determinations of glucose levels on different days were ≥7.0 mmol/l (126 mg/dL). In doubtful cases of diabetes, such as the characteristic clinical picture of diabetes and a fasting plasma glucose within 6.6–7.7 mmol/l, an OGTT was performed. An OGTT ≥11.1 mmol/l was diagnosed as type 2 DM. In addition, type 2 DM was considered when HbA1c was ≥6.5 % (according to the recommendations of the American Diabetes Association).

All of the patients underwent blood glucose measurement at admission to hospital. Moreover, the glycemic profile, including measurement of blood glucose levels in the early night and morning (3 a.m. and 5 a.m.) was assessed in 77 patients with diabetes on days 6–10 of the course of MI to monitor the efficiency of blood-glucose-lowering therapy and to detect hypoglycemic episodes. During the in-hospital period, patients were treated according to the Russian Society of Cardiology guidelines for diagnosis and treatment of patients with acute ST-segment elevation MI (2007).

The endpoints of prognosis at 1 year were overall and cardiovascular mortality, recurrent MI, progression of angina pectoris, decompensated heart failure, acute stroke, hospitalization for acute coronary events, and urgent repeated revascularization. Achievement of these endpoints was evaluated as an unfavorable outcome. The survival rate and time to onset of adverse events were assessed.

### Assays

Blood serum and plasma were collected. Serum was separated from venous blood by centrifugation at 3000 × *g* for 20 min and stored at −70 °C. Glucose levels were measured using a standard Thermo Fisher Scientific test system (Thermo Fisher Scientific Oy, Vantaa, Finland) with a Konelab 30i biochemistry analyzer (Thermo Fisher Scientific Oy).

### Statistical analysis

The data were processed using the statistical software package Statistica 6.0 (InstallShield Software Corp., Chicago, IL, USA). Results are presented as the median (Me) and 25 % and 75 % quartiles of the Me (Q1;Q3). Time to onset of adverse events was measured using the Cox regression model. *p* < 0.05 was considered statistically significant.

## Results

Blood glucose levels at admission were measured in 520 out of 529 patients. A total of 99 (19.03 %) patients had type 2 DM, of whom 11 (11.11 %) had newly diagnosed DM within the in-hospital period. DM was diagnosed in four (36.36 %) patients according to the results of the OGTT and in seven (63.64 %) patients based on glucose levels determined within a day. Patients with diabetes were significantly older than non-diabetic patients (*p* = 0.002). Female patients with DM (63.0 %) were more prevalent than female patients without DM (28.67 %, *p* = 0.001). There was a higher rate of obesity (75.0 %), as well as hypertension (96.0 %), angina pectoris (64.0 %), previous MI (38.0 %), congestive heart failure (19.0 %), and hypercholesterolemia (20.0 %), in patients with DM than in those without DM (*p* < 0.05). Patients with diabetes had a significantly higher average Thrombolysis In Myocardial Infarction risk score (5.0) than those without DM (3.0, *p* = 0.001). The in-hospital mortality rate was higher in patients with diabetes (21.05 %) than in non-diabetic patients (12.57 %, *p* = 0.008).

To evaluate the effects of blood glucose levels on in-hospital mortality, all patients, regardless of diabetic status, were assigned to three groups according to blood glucose levels at admission. One group included MI patients with euglycemia on admission (blood glucose levels <7.0 mmol/L), a second group included patients with moderate hyperglycemia (7.1–9.0 mmol/L), and a third group included patients with severe hyperglycemia (>9.0 mmol/L). Blood glucose levels at admission as a criterion for group assignment was adopted from the clinical trial the Japanese Acute Coronary Syndrome Study, which aimed to assess the prognostic value of hyperglycemia [6].

The clinical and demographic data of MI patients with and without DM are shown in Tables 1 and 2.

**Table 1:** Clinical and demographic characteristics of diabetic patients with myocardial infarction according to blood glucose levels

Variable	All diabetic patients	Diabetic patients			
		Euglycemia	Moderate hyperglycemia	Severe hyperglycemia	*p*
Number of patients, n (%)	99	5 (5.05)	9 (9.09)	85 (85.85)	
Age, years	68.0 (59.0-74.0)	68.0 (63.0-76.0)	63.0 (53.0-71.0)	68.0 (59.0-73.0)	>0.05
Males, n (%)	37 (37.37)	1 (20.0)	3 (33.33)	33 (38.82)	>0.05
AH, n (%)	95 (95.95)	5 (100.0)	8 (88.88)	82 (96.47)	>0.05
Killip II – IV, n (%)	40 (40.40)	3 (60.0)	3 (33.33)	34 (40.0)	>0.05
Time before admission, hour	4.72 (2.4;7.7)	4.0 (1.8;10.1)	9.3 (5.4;15.6)	3.6 (1.4;6.7)	>0.05
Reperfusion, n (%)	52 (52.52)	4 (80.0)	4 (44.44)	44 (51.76)	>0.05
Thrombolysis, n (%)	19 (19.19)	1 (20.0)	1 (11.11)	17 (20.00)	>0.05
PCI, n (%)	42 (42.42)	3 (60.0)	3 (33.33)	36 (42.35)	>0.05
Admission blood glucose, mol/L	15.0 (11.30-20.0)	6.20 (6.20-6.40)	8.30 (7.43-8.50)	13.80 (7.04-21.03)	P_2–3_ < 0.0001*

*Mann–Whitney test

**Table 2:** Clinical and demographic characteristics of non-diabetic patients with myocardial infarction according to blood glucose levels

Variable	All non-diabetic patients	Non-diabetic patients			
		Euglycemia	Moderate hyperglycemia	Severe hyperglycemia	*p*
Number of patients, n (%)	421	183 (43.46)	141 (33.49)	97 (23.0)	
Age, years	61.0 (53.0-72.0)	58.0(51.0-69.0)	61.0 (55.0-73.0)	69.0(61.0-76.0)	P_1–2_ < 0.0001*
					P_1–3_ < 0.0001*
Males, n (%)	300 (71.25)	143 (78.14)	98 (69.50)	59 (60.82)	P_1–2_ = 0.05**
					P_1–3_ = 0.001**
AH, n (%)	367 (87.17)	165 (90.16)	116 (82.26)	86 (88.65)	P_1–2_ = 0.02**
Killip II – IV, n (%)	94(22.32)	22 (12.02)	28 (19.85)	44 (45.36)	P_1–2_ = 0.03**
					P_1–3_ < 0.0001**
					P_2–3_ < 0.0001**
Time before admission, hour	3.1 (1.9; 6.6)	3.0 (1.8; 8.0)	3.1 (2.0; 6.2)	3.4 (2.0; 5.5)	>0.05
Reperfusion, n (%)	250 (59.38)	102 (55.73)	88 (62.41)	60 (61.85)	>0.05
Thrombolysis, n (%)	72 (17.10)	18 (9.83)	34 (24.11)	20 (20.61)	P_1–2_ = 0.0005**
					P_1–3_ = 0.01**
PCI, n (%)	213 (50.59)	93 (50.81)	69 (48.93)	51 (52.57)	>0.05
Admission blood glucose, mol/L	7.20 (6.04-8.72)	5.90 (5.21-6.4)	7.80 (7.37-8.22)	10.60 (6.33-12.70)	P_1–3_ = 0.003**
					P_2–3_ = 0.001*

*Mann–Whitney test; **Fisher’s test

The highest rate of in-hospital mortality (20.0 %) was found in patients with glycemia on admission, corresponding to euglycemia, whereas the lowest rate of mortality rate (11.1 %) was found in patients with moderate hyperglycemia.

Non-diabetic MI patients with euglycemia on admission demonstrated the lowest rate of in-hospital mortality (2.7 %), whereas the highest rate was observed in the group of patients with severe hyperglycemia (20.6 %, *p* < 0.001). Therefore, an increase in blood glucose levels by 1 mmol/L in MI patients without diabetes at admission was associated with a 10.1 %-fold increase in in-hospital mortality.

Comparative analysis of blood glucose levels at admission between patients with a different 1-year prognosis showed that non-diabetic patients with a poor prognosis had significantly higher blood glucose levels than non-diabetic patients. Patients with diabetes with a poor prognosis also had higher blood glucose levels than non-diabetic patients, but this was not significant.

Fasting glucose levels, measured on days 10–14 from the onset of MI, were similar in patients with DM and those without DM with a poor prognosis and high blood glucose levels (*p* > 0.05). Assessment of the glycemic profile, including measurement of blood glucose levels in the early night and morning hours (3 a.m. and 5 a.m.) showed that 27 (35.07 %) patients with diabetes suffered from hypoglycemic episodes (blood glucose levels <3.5 mmol/L).

Initiated hypoglycemic therapy, including administration of insulin, was not significantly different between the groups of patients with and without episodes of hypoglycemia. Insulin was administered in five (18.52 %) patients with hypoglycemic episodes and in seven (14.00 %) patients without hypoglycemia.

Notably, in MI patients, hypoglycemic status did not affect the number of in-hospital complications. However, patients with hypoglycemia reported more frequent (*p* > 0.05) development of left ventricular aneurysm and early post-infarction angina. There were no significant differences in the mortality rate and the incidence rate of ventricular arrhythmias in patients with and without hypoglycemia.

With regard to significant hypoglycemic episodes, as a marker of 1-year unfavorable prognosis in MI patients, patients with hypoglycemia were more likely to have decompensated heart failure and new coronary acute events (recurrent MI and progressive angina) during the 1-year follow-up period (Table 3). Therefore, hypoglycemia appeared to have a higher predictive value than a clinical value. Overall, there were no cases with a favorable prognosis among patients with hypoglycemia during the 1-year follow-up period. Importantly, patients with hypoglycemia had a short time to onset of adverse events (Table 4).

**Table 3:** Prognostic significance of hypoglycemic episodes

Endpoints (after 1 year)	Patients with hypoglycemia, n (%); n = 27	Patients without hypoglycemia, n (%); n = 50	*p*
Death	4 (14.82 %)	5 (10.00 %)	≥0.05
Recurrent MI	8 (29.63 %)	10 (20.00 %)	0.034
Decompensated CHF	9 (33.33 %)	9 (18.00 %)	0.000
Unstable angina	11 (40.74 %)	6 (12.00 %)	0.000

**Table 4:** Time to onset of adverse events in diabetic patients with MI and hypoglycemic episodes

Groups of patients	Patients without adverse events, n	Time-to-onset, Me, the 25th and 75th percentiles	95 % CI
with hypoglycemia	0	2 (1–6) month	1.98 - 4.73
without hypoglycemia	6 (10.2 %)	3 (1–6) month	2.71 - 5.05

## Discussion

The current findings are consistent with the results of previous registries, suggesting 14.3 – 40.9 % prevalence of DM in patients with acute myocardial infarction according to the national population characteristics [6, 7]. However, all registries show a significant increase in the in-hospital mortality rate in diabetic patients with myocardial infarction, compared with patients without carbohydrate metabolism disorders. The GRACE registry reported that patients with ST-segment elevation myocardial infarction and previously diagnosed diabetes demonstrated 11.7 % in-hospital mortality, whereas patients without carbohydrate metabolism disorders had 6.4 % mortality rate [6]. The results of the GulfRACE register also indicated a significant increase in the in-hospital mortality rate among diabetic patients with acute coronary syndrome compared with patients without diabetes (4.4 % vs. 3.4 %, respectively, *p* < 0.01) [7, 8].

The present study showed different relationships between blood glucose levels at admission and in-hospital mortality in MI patients with and without diabetes. Therefore, blood glucose levels <7.0 mmol/L at admission in MI patients without diabetes were associated with the lowest in-hospital mortality rate, and linearly increased with increased blood glucose levels. The lowest rate of in-hospital mortality was found in patients with moderate hyperglycemia. A higher rate of in-hospital mortality in this group of patients was associated with severe hyperglycemia and euglycemia. Notably, severe hyperglycemia on admission was associated with increased in-hospital mortality in MI patients with and without DM.

When analyzing the association between diabetic patients with MI and euglycemia on admission to hospital and high in-hospital mortality, there is a negative effect of hypoglycemia on the prognosis in these patients [9]. However, patients with diabetes tend to adapt better to higher blood glucose levels than to normal levels. Therefore, euglycemia (diagnosed on admission) may be regarded as relative hypoglycemia affecting the prognosis. Consequently, chronic hyperglycemia (estimated on admission and on days 10–14 of the in-hospital period) could be an important marker of poor prognosis in MI patients with and without DM.

Comparative assessment of the effect of glycemic status on the prognosis in patients with MI should consider postprandial glucose levels, which play an important role in the development of adverse cardiovascular events [10, 11]. The DECODE study [12], which assessed the risk of death under different variants of hyperglycemia, showed that the postprandial glucose level is an independent risk factor, with higher prognostic significance than glycated hemoglobin levels. Several studies have suggested that after-meal high blood glucose levels are more important for the risk assessment of cardiovascular complications in diabetic patients than fasting glucose levels [12]. Therefore, assessment of the effects of acute and chronic hyperglycemia on induction of oxidative stress in patients with DM suggests that its main triggering mechanism is acute deviations in blood glucose levels, but not long-term chronic hyperglycemia (or small fluctuations in postprandial blood glucose) [13]. Postprandial hyperglycemia in patients with DM is associated with reduced myocardial perfusion caused by microvascular abnormalities. We have previously shown a positive effect of adequate glycemic control [14].

In general, hyperglycemia on admission in ACS patients is a major predictor of survival and increased risk of development of cardiovascular events, regardless of diabetic status [1]. Moreover, the present study showed that hyperglycemia on admission to hospital, as an indicator reflecting the degree of oxidative stress in acute coronary events, significantly affects immediate and long-term prognosis in patients with MI. However, the majority (74.2 %) of patients with DM had high levels of postprandial and fasting glucose (i.e., presence of chronic hyperglycemia), which affected the prognosis during the in-hospital period. Notably, the prognostic value of hyperglycemia did not change during the whole in-hospital period [15]. Nevertheless, previous studies have shown an increase in the mortality rate in patients with hypoglycemia and MI [15–17].

Taking into consideration the adverse effect of hypoglycemic episodes on the long-term prognosis in MI patients with DM, spontaneous hypoglycemia might be a marker of the severity of disease and adverse outcomes or an independent predictor of a poor prognosis. Further studies are required to determine the role of hypoglycemia as a mediator of adverse outcomes in MI patients.

## Conclusions

Hyperglycemia, assessed in patients with MI with and without DM when they are admitted to hospital, affects the short and long-term prognosis. Hyperglycemia and the in-hospital prognosis show a linear relationship in patients without DM. The presence of hypoglycemia in patients with DM does not affect the in-hospital prognosis, but enables determination of patients with an unfavorable course in the postinfarction period.

## Abbreviations

MI: myocardial infarction
ACS: acute coronary syndrome
DM: diabetes mellitus
OGTT: oral glucose tolerance test
